# Sclerema Neonatorum Treated Successfully with Parenteral Steroids: An Experience from a Resource Poor Country

**DOI:** 10.1155/2017/4836142

**Published:** 2017-05-11

**Authors:** Sandeep Shrestha, Nagendra Chaudhary, Sujit Koirala, Ruchi Gupta

**Affiliations:** Department of Pediatrics, Universal College of Medical Sciences, Bhairahawa 32900, Nepal

## Abstract

Sclerema neonatorum is a form of panniculitides characterized by diffuse hardening of subcutaneous tissue with minimal inflammation. It usually affects ill and preterm neonates. Prognosis is usually poor in many cases despite aggressive management. Various treatment modalities (antibiotics, intravenous immunoglobulin, steroids, and exchange transfusion) have been explained in literature. Steroids due to its easy availability and low cost can prove to be lifesaving in such cases, especially in resource poor countries. Here, we report a case of sclerema neonatorum in a one-week preterm baby treated successfully with parenteral steroids and antibiotics.

## 1. Introduction 

Sclerema neonatorum (SN) is a rare and severe skin condition presenting with generalized hardening of subcutaneous tissues [1]. It is mostly seen in ill preterm neonates during the first week of life but may develop immediately postpartum or after several weeks of life [2]. The skin is bound to underlying subcutaneous tissues including bone and muscles and therefore cannot be pitted or picked up. The process of hardening usually begins at the buttocks, thigh, or trunk and then progresses diffusely to involve other parts of the body. Fat free areas such as palms, soles, and genitalia are usually spared. Movement, feeding, and respiration are affected due to hardening in such cases. Prognosis is usually poor despite aggressive interventions [3–6].

## 2. Case Summary

A preterm neonate was brought on third day of life at our NICU with chief complaints of poor feeding, lethargy, and diffuse hardening of the skin. It was an unbooked case; however, mother had 3 antenatal visits at local primary health center. Mother was 22-year-old primigravida with blood group of A-positive. First and second trimesters were uneventful. Iron, folic acid, and calcium supplementation was taken for one month during the pregnancy which she left on her own. She also received two doses of tetanus toxoid vaccinations during her second trimester of pregnancy. Routine antenatal ultrasonography scan for fetal wellbeing was not done. Viral markers (HBs Ag, Anti-HCV, and HIV) were also not done. At 33^+4^ weeks of gestation (as per LMP), she had lower abdominal pain with nonfoul smelling vaginal discharge. She delivered a male neonate at home with help of a local birth attendant after 24 hours of vaginal discharge. Cord was cut with clean blade and umbilical stump was tied with sterile cotton bandage. Baby cried immediately after birth. He was fed with glucose water solution as mother's milk secretion was scanty. The first day was uneventful; however, on the second day, mother noticed that baby was lethargic and was not taking expressed breast milk. On routine care, she felt unusual hardening around bilateral thighs and buttocks. Gradually, hardening of skin progressed to trunk, neck, and cheek muscles. The baby could hardly open his mouth for feeding. On Day 3 of life, baby was brought to our NICU.

At presentation, baby was lethargic and hypothermic. His weight was 1550 grams and axillary temperature was 35.2°C. Random blood sugar (RBS) was 64 mg%. On examination anterior fontanelle was at level. There were no dysmorphic features or any obvious congenital defects. There was diffuse hardening of skin involving lower limbs, trunk, neck, upper limb, and facial muscles. Heart rate was 156 beats/min, respiratory rate was 44/min, oxygen saturation was 94% at room air, and capillary refill time was 2 seconds. Other systemic examinations were normal. A provisional diagnosis of early onset neonatal sepsis (EONS) with diffuse sclerema was made. Child was kept under radiant warmer. Intravenous fluids (1/5 NS + 5% dextrose + 20 meq/L of KCl) were started along with empirical antibiotics for early onset neonatal sepsis (ampicillin plus gentamycin).

Total leukocyte count at admission was 10000/mm^3^ with neutrophil of 76%. Platelet count was 100000/mm^3^. Serum sodium was 142.7 mmol/L, serum potassium was 4 mmol/L, urea was 53 mg%, and creatinine was 0.9 mg%. Total serum calcium was 9.2 mg/dL. C-reactive protein (CRP) was positive (30 mg/L) with micro-ESR, 15 mm (in 1st hour). Blood culture and sensitivity were sent which did not grow any organisms. CSF studies were also normal. The child did not respond properly to the abovementioned treatment during the first 48 hours of admission; rather, sclerema progressed further. Hypoglycemia was noted at 30 hours of life [RBS, 28 mg/dL by glucometer and corresponding laboratory blood glucose (measured by glucose oxidase method) was 32 mg/dL]. Intravenous 10% dextrose at the rate of 2 mL/kg was given. Glucose infusion rate (GIR) was increased from 6 mg/kg/min to 8 mg/kg/min. At 36 hours of life, child developed next episode of hypoglycemia. RBS as measured by glucometer was 34 mg/dL and so GIR was increased to 10 mg/kg/min. RBS was taken every hourly till child was euglycemic (RBS > 45 mg/dL) and then successive RBS was taken every 6 hours. Child was kept at GIR of 10 mg/kg/min for 24 hours. There were no further episodes of hypoglycemia. Then GIR was decreased every 6 hours by 2 mg/kg/min and in next 12 hours child was under regular maintenance fluid with GIR of 4 mg/kg/min.

In the view of progressing sclerema and hypoglycemic episodes, complete blood count along with blood culture and CRP were resent and antibiotics upgraded to second line (vancomycin plus piperacillin tazobactam) as per our NICU protocol. Repeated complete blood count after 48 hours showed TLC of 38700/mm^3^ with neutrophil of 60%. Platelet count was 45000/mm^3^. CRP was 45 mg/L. Repeated blood culture also did not grow any organisms. Despite second-line antibiotics, baby's condition was deteriorating.

We performed a literature search regarding the possible management of worsening sepsis with sclerema neonatorum. Both intravenous immunoglobulin (IVIG) and exchange transfusion (ET) were found to be of some benefits. Decision to treat with IVIG was considered but patient party could not afford IVIG neither did they give the consent for ET. We had one option left with steroids (ACTH or hydrocortisone) which could be used in cases of sclerema neonatorum with little benefit. As ACTH was not available locally, we started parenteral hydrocortisone at the dose of 50 mg/m^2^/day in three divided doses. Sclerema started improving within 24 hours of parenteral hydrocortisone. After 72 hours of therapy, his sclerema almost resolved except over localized areas on anterior aspect of bilateral lower limbs. His activity improved significantly and there was no stiffness on mouth opening. Expressed breast milk was started through katori-spoon initially at 20 mL/kg/day. Feed was gradually increased and by 10th day of life, child was on full breast feeding. Repeat CBC at 10th day of life showed TLC of 14200/mm^3^ with neutrophil of 55% and platelet count was 240000/mm^3^. Repeated CRP was negative. Child received IV hydrocortisone for total of 5 days and was stopped. IV antibiotics were given for total 10 days. Child was discharged on 17th day of life. Child was followed up at 30th day of life. His sclerema improved completely and was gaining weight adequately.

## 3. Discussion

Sclerema neonatorum (SN) is a rare skin condition, the major manifestation of which is generalized hardening of skin and subcutaneous tissue of infants [1]. The first clinical description of SN was given by Underwood in early 18th century when he described it as “skinbound” [7]. There are other skin conditions (subcutaneous fat necrosis of newborn, scleredema) presenting similarly as hardening of skin which may cause diagnostic difficulties [2]. Sclerema neonatorum usually affects premature neonates with generalized hardening of skin. It is associated with sepsis and prognosis is usually grave [3–6]. Subcutaneous fat necrosis of newborn (SCFN) usually affects postmature neonates with localized areas of hardness. It is associated with birth asphyxia and trauma. Scleredema affects premature neonates with generalized firm pitting edema unlike SN where there is no edema. Prognosis of both SCFN and scleredema is good and both heal spontaneously. The definite diagnosis is usually made by biopsy [8–10].

In our case, we made a diagnosis of SN on the basis of clinical grounds as proposed by Hughes and Hammond [3]. The neonate was preterm, hypothermic with features and risk factors for sepsis with generalized hardening of skin with no pitting edema. However, we could not perform skin biopsy as parents denied. Neonates have high ratio of saturated to unsaturated fatty acids unlike adults. Therefore, neonatal fat has high melting point and low solidification point that tends to harden with fall in temperature. Sepsis causes circulatory collapse with fall in body temperature as in our case causing generalized hardening of skin [11, 12].

Previous case reports and case series show that SN usually affects preterm neonates within first week of life. It may be associated with other neonatal comorbidities like sepsis, hypothermia, hypocalcaemia, and congenital anomalies. There may be associated maternal complications like preeclampsia, eclampsia, and prolonged rupture of membranes (PROM), placenta previa, and hyperpyrexia [2, 4–6]. In our case, the affected neonate was preterm associated with sepsis and hypothermia with no obvious congenital anomalies. There was prolonged rupture of membranes. The process of generalized skin hardening started initially around the buttocks and thigh on 2nd day of life and gradually progressed to other sites over next 2 days.

Management of SN involves intensive therapy for underlying sepsis and correction of fluid and electrolyte imbalance [2]. Despite aggressive management, prognosis is usually poor. Steroids have been used as an adjunctive therapy. Steroid use alone has not improved the survival; however, it can limit the spread of skin hardening in SN. ACTH or hydrocortisone or combination of both has been used in SN with mixed results [13–17]. Levin et al. reported 25 preterm neonates with sclerema neonatorum of which 11 neonates (group A) were treated with steroids while 14 neonates (group B) were treated with antibiotics and supportive therapy without steroids. In group A, neonates received hydrocortisone 50 mg intramuscular followed by 25 mg every 8 hours for 5 days. Only one neonate in group A survived while 3 in group B survived. The mortality was high in both groups and there was no significant difference between neonates treated with or without steroids [13]. Another case of sclerema neonatorum treated successfully with steroid has been reported by Sondergaard and Nielsen [14]. The child was 31 days old delivered vaginally at term and presented with sepsis. Child was started on penicillin therapy but despite antibiotic therapy, he deteriorated. On second day of admission, child developed hardening of lower limbs which gradually progressed to involve trunk and upper limbs. Child was started on ACTH therapy 5 mg 12 hourly and continued for 9 days. Sclerema and general condition of child improved.

Similarly, IVIG and exchange transfusion (ET) have been used to improve the outcome of SN. ET has shown improved outcomes in neonates with SN. ET enhances immunoglobulin and complement levels and improves survival especially in preterm neonates [18–21]. IVIG also enhances humoral and cellular immunity but has not improved the survival in SN [22–24]. Sadana et al. studied the effect of exchange transfusion in septic neonates with sclerema. It was a randomized controlled trial involving 40 neonates of which only 20 cases received ET. Mortality was 50% in study group and 90% in controls and the result was statistically significant [18]. Another study by Narayanan et al. comprised 60 neonates with severe septicemia with sclerema. The infants were divided into 3 groups each consisting of 20 neonates. All of them received standard treatment including antibiotics. Neonates in group II were given a simple transfusion (whole blood of 20 mL/kg) and those in group III received exchange transfusion (160–190 mL/kg of fresh blood). Morbidity and mortality were significantly less in neonates who received exchange transfusion in addition to conventional treatment (*p* < 0.05) [19]. A recent Cochrane Database of systematic reviews did not find any significant change in mortality and morbidity between neonates with severe sepsis treated with or without IVIG [24].

In our case, we opted for ET because of its promising results and feasibility in our setting. However, parents denied the consent for ET. IVIG could not also be given due to its unaffordability by the parents. Finally, we decided to start steroids. As ACTH was not available, we started IV hydrocortisone at doses of 50 mg/m^2^/day in three divided doses. Within 24 hours of starting hydrocortisone, sclerema started improving. By 72 hours, sclerema almost subsided. We gave hydrocortisone for total of 5 days without tapering.

## 4. Conclusion

Sclerema neonatorum is a rare pathological condition usually affecting preterm neonates with high mortality. In low resource settings like Nepal, steroids can be regarded as an important treatment modality in SN due to its easy availability and low cost where IVIG and exchange transfusion may not be feasible or affordable. Further multicentric trial studies on larger scales are necessary to come into conclusion regarding steroid effectiveness in sclerema neonatorum.
